# Supplementary material for: Hierarchical AI enables global interpretation of culture plates in the era of digital microbiology
Source: Nat Commun. 2023 Oct 28;14:6874. doi: 10.1038/s41467-023-42563-1 (PMC10613199; doi:10.1038/s41467-023-42563-1)
Supplement: Supplementary file 1 — Supplementary Information [file 41467_2023_42563_MOESM1_ESM.pdf]

Hierarchical AI enables global interpretation of culture plates in the era of digital microbiology

Alberto Signoroni[1,2,*,†], Alessandro Ferrari [3,4,*], Stefano Lombardi [1,3], Mattia Savardi[1,2], Stefania Fontana [3], Karissa Culbreath [5]

Supplementary Information

Supplementary Notes

Supplementary Note 1: *Single colony CNN architecture details*
The network for single colony identification is composed of:

- input layer: 3 color channel 3x128x128 image;
- 1st convolutional layer: 20 features maps with filter size 5x5;
- 2nd conv. layer: 50 features maps with filter size 5x5;
- 3rd conv. layer: 100 features maps with filter size 4x4;
- 4th conv. layer: 200 features maps with filter size 4x4;
- fully connected layer, 500 hidden units;
- soft-max output layer with 32 output units.

Unlike standard architectures, like the one used in[1], where non-saturating non-linearities ReLUs[2] were adopted, Leaky ReLUs were chosen[3], since they allow the learning to progress even when the input of the activations is negative, slightly improving results. The PReLU[4] was also tested but performed slightly worse, probably due to increased overfitting. The outputs of the Leaky ReLU activations are normalized using Local Response Normalization (LRN)[5]. A non-overlapping max-pooling layer with stride equal to 2 is added to increase distortion invariance and reduce the number of parameters to be learned. Dropout[6] is applied to the connections from the top of the last convolutional layer to the last softmax layer, to reduce overfitting and prevent co-adaptation of feature detection on the fully connected stage. The dropout rate has been cross validated to 0.75. Networks weights are initialized with Xavier, coherently to what suggested in[7], and the training was performed with Stochastic Gradient Descent with batch size 64 and momentum set to 0.9. For regularization, weight decay was set to 0.0005. Learning rate is initialized to 0.01 and decreased by 0.01% at each iteration and halved after 50,000 iterations.
The square image segments representing isolated colony from the *colony-level clinical dataset* were all scaled to 128×128-pixel size and flipped so as to augment dataset cardinality. A fixed margin is introduced on the longer side before rescaling to preserve valuable morphometric information about the original colony size, which can be learned by the network. Interestingly, experimental results show that other configurations in which this information is lost (e.g., when the colony always occupies the entire area), lead to poorer performance.

Supplementary Note 2: *Siamese CNN architecture and training details*

[1] Department of Information Engineering – University of Brescia, Brescia, Italy
[2] Dept. of Medical and Surgical specialties, Radiological Sciences and Public Health – Univ. of Brescia, Brescia, Italy
[3] Copan WASP – Brescia, Italy
[4] NVIDIA – Munich, Germany
[5] Department of Infectious Disease, Tricore Laboratories – Albuquerque, New Mexico, USA
[*] These authors contributed equally to this work
[†] Corresponding Author: alberto.signoroni@unibs.it

The Siamese CNN (S-CNN) is similar in structure with respect to the single colony identification network. Each convolutional neural network has 2 convolutional layers, 2 fully connected layers and one output layer. The size of the output layer determines the dimension of the target space. On top of every layer, there are PReLU non-linearities[4]. A single max-pooling layer with stride length 1 is placed on top of the first convolutional layer. There are no other pooling layers, since in measuring similarity, too much distortion invariance can be detrimental[8]. As a result, the size of the convolutional feature maps before the fully connected layers are quite large. Even so, this configuration of the model performed the best on cross-validation set. Fully connected layers have dropout set to 0.5, to reduce overfitting[6].
The dataset creation for the S-CNN training is done so that only if the represented colonies come from the same plate and belong to the same strain, they can form genuine pairs (and their label is set to 1); otherwise, if they belong to different strains, they are classified as impostor pair with a label set to 0. Pairs in both the training and validation sets are equally split between genuine and impostor.
The contrastive loss layer[9] for the S-CNN training is defined as:

$$E_w = \frac{1}{2N}\sum_{n=1}^{N} y * \|x_n - xp_n\|_2 + (1-y) * \max\left(1 - \|x_n - xp_n\|_2, 0\right)^2 \quad (1)$$

where x and xp are the inputs to the contrastive layers, while y is 1/0 if the nth-pair of images is a genuine/impostor pair. Network weights and bias values are initialized in the way described above for the CNN operating on single colonies. Training was performed with the ADAM gradient-based optimization method[10]**,** with batch size 64. For regularizing, weight decay was set to 0.0005.

Supplementary Note 3: *Clustering metrics*
Homogeneity, completeness, and *v-measure* are three widely used metrics to evaluate the performance of clustering algorithms[11]. Homogeneity measures the extent to which each cluster contains only data points that belong to a single class. In other words, homogeneity measures the quality of the clustering results based on how well the clusters match the true class labels of the data points. Completeness measures the extent to which all data points that belong to a given class are assigned to the same cluster. In other words, completeness measures the quality of the clustering results based on how well the true class labels of the data points match the cluster assignments. *v-measure* is a harmonic mean of homogeneity and completeness. It provides a single measure that balances the trade-off between these two metrics. Achieving perfect homogeneity requires that all colonies within a cluster belong to the same strain. Conversely, an algorithm that groups colonies from different strains within the same cluster will have suboptimal homogeneity. However, if colonies from the same strain are partitioned into multiple subsets without mixing them with other strains, the resulting clustering will still exhibit good homogeneity. In contrast, if all colonies from a given strain are clustered together, the algorithm will have good completeness. A clustering algorithm that assigns a cluster to each colony will exhibit maximum homogeneity but null completeness, whereas an algorithm that assigns all colonies to the same cluster will exhibit maximum completeness but null homogeneity. Therefore, prioritizing homogeneity at the expense of completeness can lead to over-partitioning, while sacrificing homogeneity to increase completeness can lead to under-partitioning. Notably, a clustering system that assigns colonies belonging to different strains to the same group would result in incorrect presumptive identification for some of them. Conversely, if colonies from the same strain are assigned to different groups, they might receive different probability scores associated with different classes, but the scores are likely to be quite similar. Therefore, it is preferable to use a clustering system that favours homogeneity over completeness to smooth classification. The best parameters for the clustering algorithm and the best performing S-CNN were jointly selected through cross-validation, comparing the resulting *v-measure* score median over the virtually mixed flora dataset portion constructed using plates from the validation set of the isolated colonies dataset. **Supplementary Table 3** shows Homogeneity, Completeness, and *v-measure* statistics for the best clustering and S-CNN combination, which resulted in a significant improvement in the strain identification performance.

Supplementary Figures

a) Clinical plates with pure growth

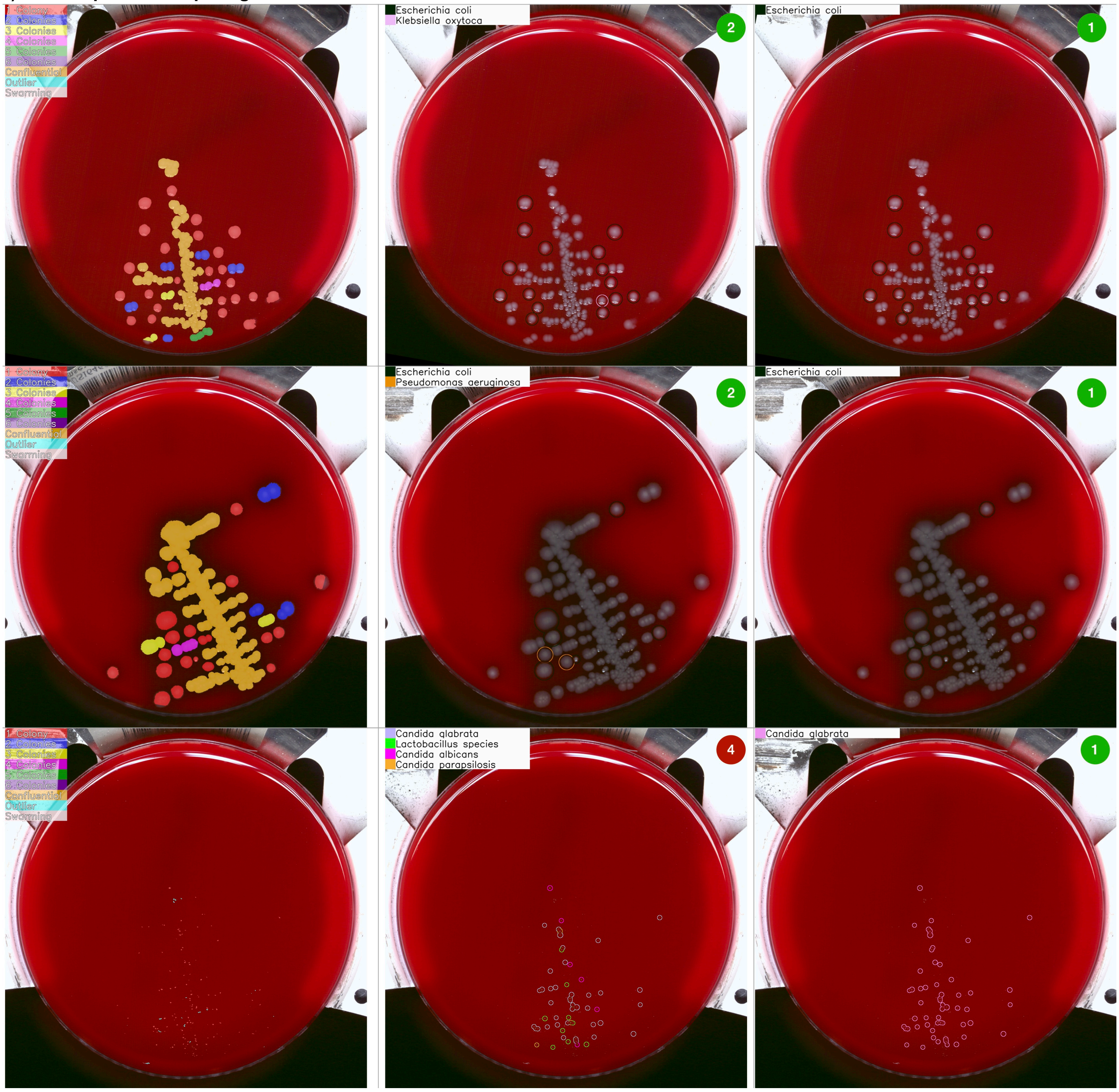

Supplementary Fig. 1 | *DeepColony* in action on clinical plates: three groups of plates are represented in subfigures **a**, **b** and **c** to exemplify the action of *DeepColony*. For each subfigure, rows represent different plates and columns different stages. On the ***left column***, the *segmentation/enumeration* maps (*level 0*) are shown. On the ***central column***, the species identified (Top-1 ID) after *single colony identification* (*level 2*) are listed in legend (random color key assignment) and visualized by contouring each good colony (from *level 1*) using the corresponding color. Similarly, the final species identified (Top-1 ID) after *context-based identification refinement* (*level 3*) are shown on the ***right column***. For the central and right columns, the round labels put on the upper right corner indicate the number of identified species and represent the final plate significance outcomes (green = significant, red = non-significant), based on *level 4* and according to the rules of **Fig.5a**. Notably, the right column indicates the actual outcomes produced by the whole *DeepColony* action, while the central column shows the hypothetical outcome produced by skipping *level 3* (i.e. where the interpretation of the plate is based only on the information generated at *level 2*). This provides a better appreciation of the effect of the contextual regularization brought by *level 3*.

a *Clinical plates with pure growth*: in this group of plates, *DeepColony* determines the pure growth of a single significant pathogen. Noticeably, comparing the hypothetical and the actual outcomes (central and right columns) it is evident how *context-based identification* (*level 3*) leads to a better defined indication of pathogens and, most importantly, to avoid losing a plate that, without *level 3*, would have been judged non-significant.

b) Clinical plates with 2-3 significant pathogens

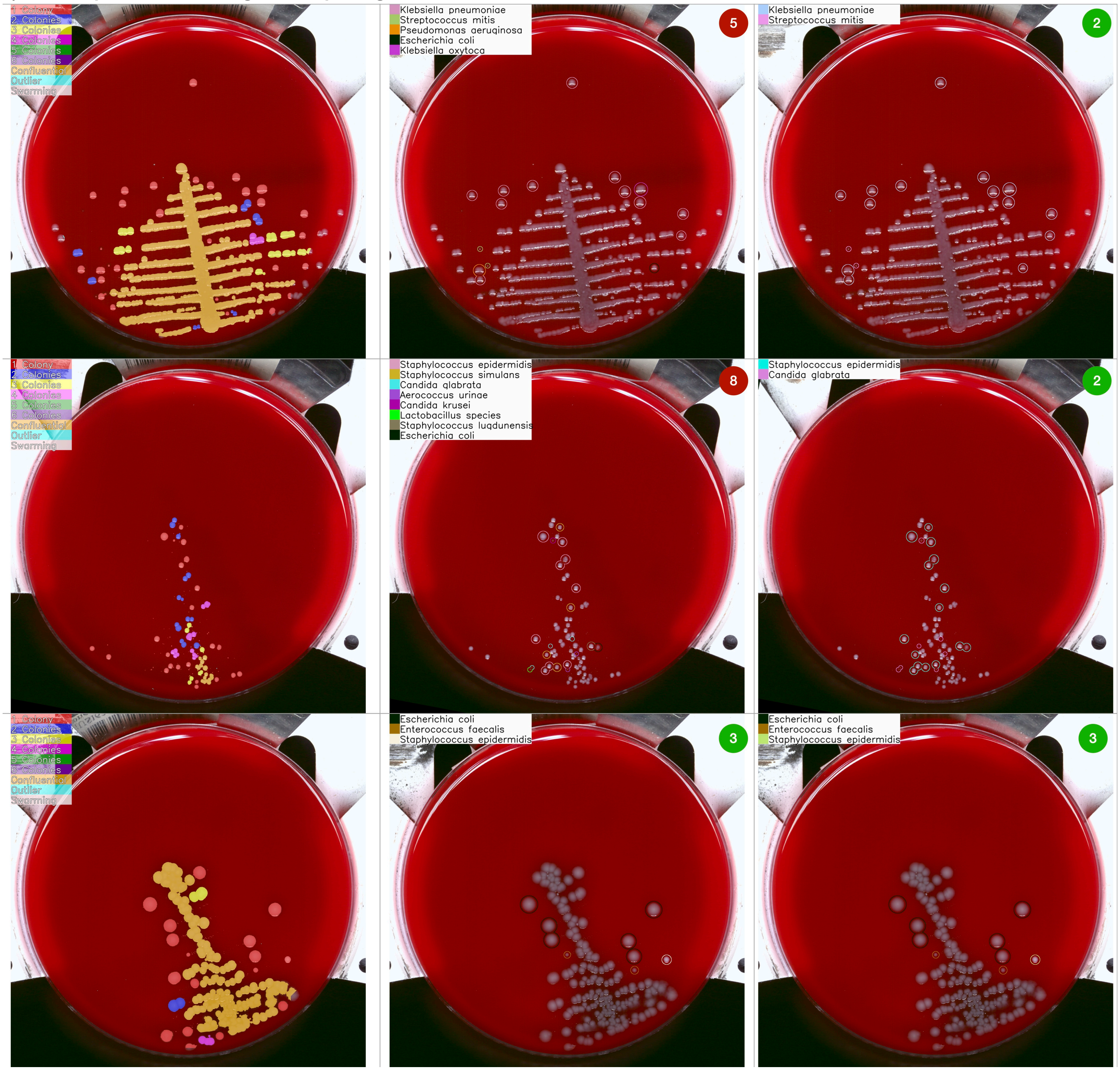

Supplementary Fig. 1 | (contd.)
b *Clinical plates with 2-3 significant pathogens*: in this group of plates, *DeepColony* determines the possibility of growth of two or three significant pathogens, with plates identified as significant, according to the plate interpretation rules of **Fig.5a**. Note how the system is both able to regularize the number of detected pathogens (avoiding false negative plate interpretations) and also to confirm the presence of multiple pathogens without reducing their number.

c) Clinical plates considered contaminated with genitourinary or skin flora

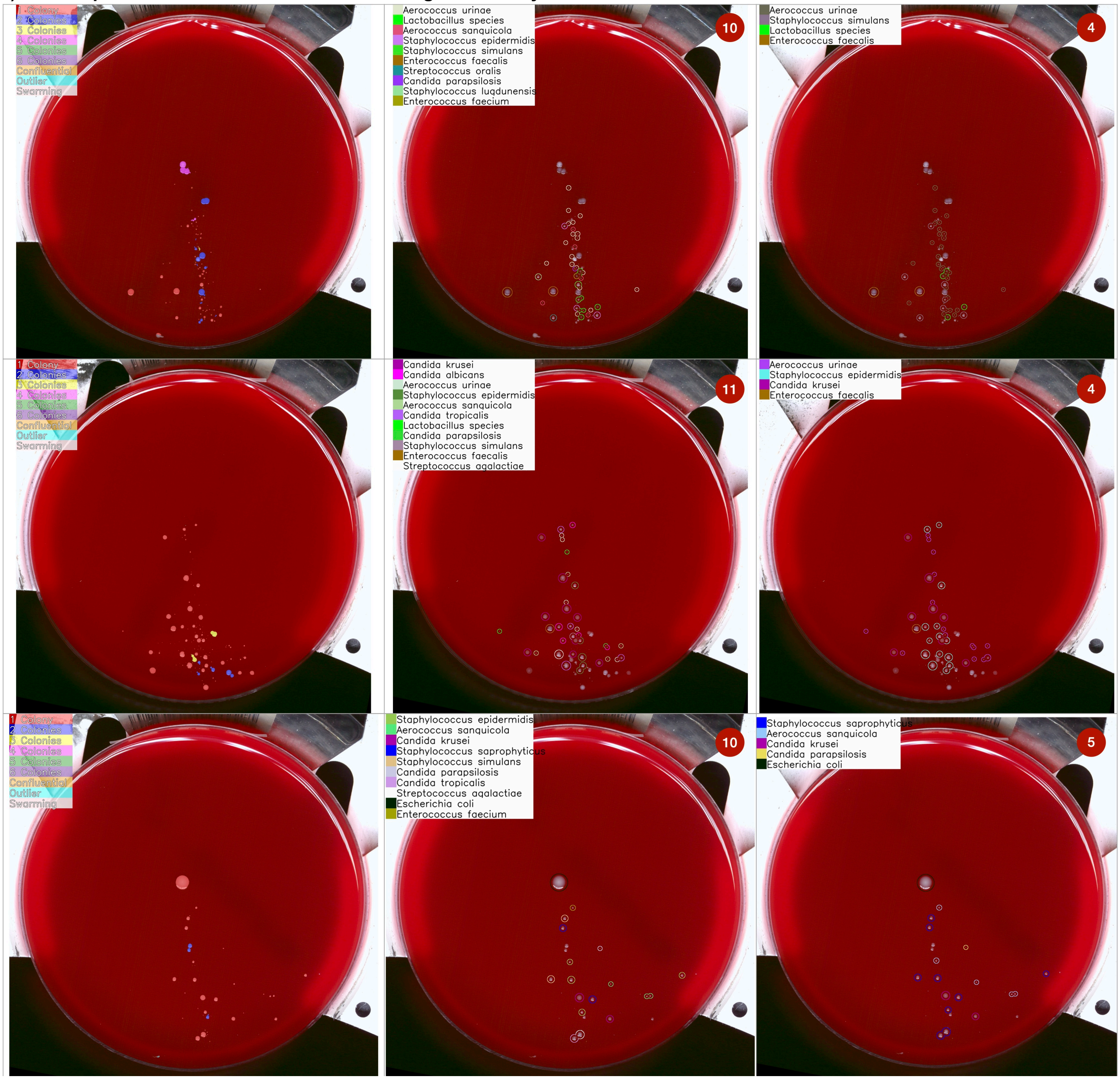

Supplementary Fig. 1 | (contd.)

c *Clinical plates considered contaminated with genitourinary or skin flora*: the cultures depicted here are determined to have growth >3 organisms at concentrations considered to be insignificant based on the laboratory rules. For this group, the *context-based identification* refinement (*level 3*) lead to a reduction of the number of suggested pathogens, without altering the non-significance interpretation, rather strengthening it.

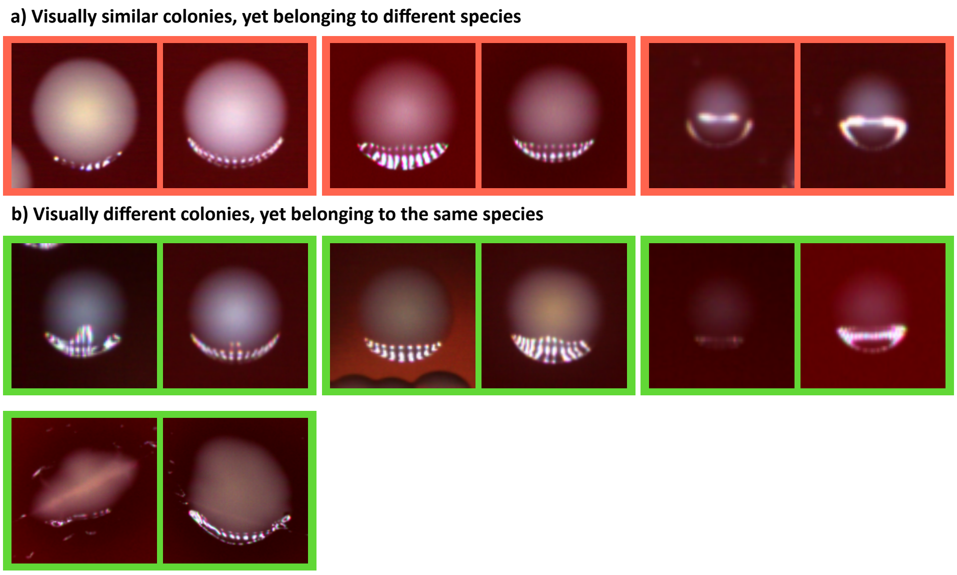

Supplementary Fig. 2 | *Inter-species similarity vs Intra-strain differences.* Visual discrimination of colonies belonging to the same or different species is a very challenging task. In fact, there are many cases in which visually similar colonies belong to different species (a), or the other way around (b). Notice that this is not only a morphology-related question, but it also depends on texture, illumination (in this case highlighting specific morphological and material aspects), and colour-related features.

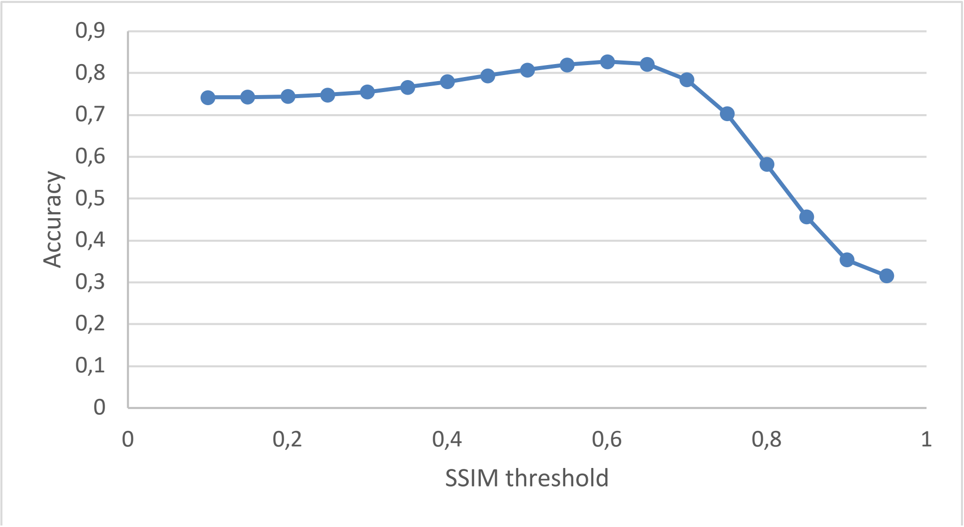

Supplementary Fig. 3 | *SSIM Accuracy.* Discrimination power between genuine and impostor pairs using SSIM features. Siamese CNN performances are at 99.1% accuracy level, while the graph shows that, even with varying the threshold, discrimination based on SSIM never goes over 83% accuracy with a dramatic penalization of SSIM values over 0.7. This is clear evidence that SSIM may not be an appropriate metric for this particular task since we are not seeking straightforward visual similarity, but a higher conceptual level of similarity, which must encompass and accommodate for different colony sizes (due to different nourishment conditions over the plate) and, most importantly, intra-strain polymorphism.

Supplementary Tables

Supplementary Table 1. Number of involved cultured plates and extracted colony images composing the colony-level dataset. Quantitative details are given for both the 32 considered pathogen species and for the 16 groups of pathogens formed according to phylogenetic criteria, morphological similarity and diagnostic relevance. Plates are from clinical samples except for numbers in round brackets indicating plates and colonies obtained by ATCC organisms' inoculation.

		Pure-flora plate images		Pure-flora plate images: testing subset		Isolated colony images		Isolated colony images: testing subset	
**Pathogen**	**Pathogen / Group of Pathogens**	**Pathogen**	**Pathogen / Group of Pathogens**	**Pathogen**	**Pathogen / Group of Pathogens**	**Pathogen**	**Pathogen / Group of Pathogens**	**Pathogen**	**Pathogen / Group of Pathogens**
Staphylococcus aureus	*Staphylococcus aureus* (coagulase +)	131 (4)	131 (4)	57 (1)	57 (1)	2478 (482)	2478 (482)	473 (9)	473 (9)
Staphylococcus epidermidis<br>*Staphylococcus simulans*<br>*Staphylococcus lugdunensis*	coagulase-negative staphylococci (CNS)	61<br>8<br>5	74	21<br>3<br>2	26	1868<br>208<br>199	2275	374<br>46<br>63	483
Staphylococcus saprophyticus	*Staphylococcus saprophyticus*	37	37	11	11	1298	1298	256	256
Streptococcus mitis<br>*Streptococcus oralis*	viridans-group streptococci	12<br>6	18	5<br>2	7	279<br>155	434	58<br>31	89
Streptococcus agalactiae	*Streptococcus agalactiae*	56 (2)	56 (2)	21	21	1537 (347)	1537 (347)	305	305
Streptococcus pyogenes<br>*Streptococcus dysgalactiae* (Group C/G)	beta-haemolytic streptococci	16 (3)<br>9	25 (3)	5 (2)<br>4	9 (2)	395 (57)<br>286	681 (57)	75 (10)<br>59	134 (10)
Enterococcus faecalis<br>*Enterococcus faecium*	*Enterococcaceae*	57<br>51 (3)	108 (3)	21<br>18 (3)	39 (3)	1418<br>1535 (50)	2953 (50)	285<br>284 (50)	569 (50)
Aerococcus urinae<br>*Aerococcus sanguicola*	*Aerococcaceae*	27<br>17	44	10<br>7	17	674<br>632	1306	133<br>124	257
Lactobacillus species	*Lactobacillaceae*	22	22	9	9	751	751	148	148
Pseudomonas aeruginosa	*Pseudomonadaceae*	61 (7)	61 (7)	29 (2)	29 (2)	1088 (152)	1088 (152)	212 (27)	212 (27)
Escherichia coli	*Enterobacteriaceae/Escherichia*	379 (2)	379 (2)	148	148	4240 (164)	4240 (164)	843	843
Citrobacter koseri<br>*Citrobacter freundii*	*Enterobacteriaceae/Citrobacter*	21<br>33	54	9<br>13	22	225<br>341	566	45<br>68	113
Klebsiella pneumoniae<br>*Klebsiella oxytoca*<br>*Enterobacter aerogenes*<br>*Enterobacter cloacae*<br>*Serratia marcescens*	*Enterobacteriaceae* KES *(Klebsiella /Enterobacter /Serratia)*	103<br>29<br>18<br>32<br>5	187	42<br>11<br>8<br>13<br>2	76	1025<br>298<br>192<br>267<br>41	1823	204<br>60<br>38<br>53<br>9	364
Providencia stuartii<br>*Proteus mirabilis*<br>*Morganella morganii*	*Morganellaceae*	5<br>23 (5)<br>12	40 (5)	2<br>10 (3)<br>4	16 (3)	39<br>256 (81)<br>157	452 (81)	12<br>49 (20)<br>21	82 (20)
Candida albicans	*Candida albicans*	62 (4)	62 (4)	27 (3)	27 (3)	2385 (99)	2385 (99)	472 (59)	472 (59)
Candida parapsilosis<br>*Candida tropicalis*<br>*Candida krusei*<br>*Candida glabrata*	non-*Candida albicans* species	6<br>27<br>10<br>10	53	2<br>10<br>5<br>4	21	268<br>1127<br>204<br>347	1946	58<br>222<br>16<br>84	380
Total		1351 (30)	1351 (30)	535 (14)	535 (14)	26213 (1432)	26213 (1432)	5180 (175)	5180 (175)

Supplementary Table 2: *Ablation studies.* We compared the proposed baseline model, which uses maxpooling layers, with models that use either strided convolution or average pooling layers. Our results show that the baseline model outperforms the other two, possibly due to its ability to reduce geometric distortion and avoid feature extraction confusion. Additionally, we tested the popular MobileNetV2 architecture: despite producing a slight (almost negligible) increase of the overall accuracy, it came at the cost of an order of magnitude increase in number of parameters and computational complexity.

Model	Accuracy change from Baseline (in percentage points)
Baseline (83,4%)	--
Strided convolution	-0.67
Average pooling	-0.45
MobileNet V2	+0.04

Supplementary Table 3. *Best performing clustering statistics.*

	Mean	Median	Standard Deviation
Homogeneity	0.79407	1.0	0.29182
Completeness	0.93863	1.0	0.12148
v-measure	0.82398	0.94428	0.25345

Supplementary Pseudocode

Algorithm: DeepColony

Input Culture plate color image: **p**[*x*,*y*]
List of *S* identifiable species (*S* = 32): ***species***[*s*] $1 \leq s \leq S$
Plate significance rules adopted by the Clinical Microbiology lab: **plate_signif_rules**(*lab*)

Outputs Species ID ranking for every good colony on the plate: ***sID***[] // vectors of max dimension=*S* (number of species)
Estimation of CFU for each strain growing on the plate: ***cfu***[] // min-max CFU count interval for each strain
Plate significance attribution for image **p**[*x*,*y*]: ***ps*** ∈ {*significant*, *non-significant*, *contaminated, NGUF*}
Suggested colonies to pick for downstream tasks (Maldi-TOF, AST,...): ***mp***[] // list of good colony indexes to pick

Functions and Convolutional Neural Networks (CNN)

map = **CnnSE** (*image*) // Produces segmentation and enumeration map from a plate image [25]

boolean = **IsGoodColony** (*image, map, segment*) // Determines if a single colony segment is a "good colony" on a plate // image by looking at its conditions (not too close to large confluent areas // or plate borders), and exploiting a distance transform computed on the // segmentation map.

id_vector = **CnnID** (*image*) // Estimates an ID confidence ranking vector from a single colony image // by a CNN trained to recognize species among ***species***[*s*], for $1 \leq s \leq S$, // according to inter-species differentiation criteria

feature vector = **SCnnEmB** (*image*) // Branch of a Siamese CNN trained to recognize intra-strain similarity // in the plate context and to Embed single colonies in a similarity space

clustering vector = **MeanShift** *(feature_matrix)* // Mean Shift algorithm (operating on a collection of features in an // embedding space) producing a cluster assignment vector

sid_vector = **SmoothID** (*ID_matrix*, *int*) // Updates (e.g. by averaging) every species-related ID probability of the // colonies in the same cluster generating new cluster-related ID ranking

minmax_vector = **EstimateIDcount** (*map, ID_matrix*) // Estimates min-max interval of CFU count on the plate for each species // detected (first ranked) in *ID_vector* for every "good colony"

value = **PlateSignificance** (*minmax_vector,* lab*_rules*) // Estimates plate significance

index = **Colony2PickIndex**(*image, map, ID_matrix, int*) // Proposes an index (among good colonies) of colony to pick // for a given species

Level 0 – Colonies segmentation and enumeration:

Input Culture plate color image **p**[*x*,*y*],
Outputs Enumeration map **e**[*x.y*]
Set of binary segments {$\mathbf{s}_n$[*x*,*y*]} 1≤*n*≤*N* // $\mathbf{s}_n$ *n*-th segment (binary connected comp.), *N* # of segments
Vector of colony counts ***c***[*n*] 1≤*n*≤*N* // *c* how many colonies in the segment

```
compute e[x.y] = CnnSE (p[x,y])
N = e.number_of_segments
for 1 ≤ n ≤ N
    s_n[x,y] = e.output_segment_mask(n)
    c[n] = e.output_segment_count(n)
end for
```

continue →

Level 1 – Good colony extraction:

```
Input p[x,y], {s_n[x,y]}, c[n]
Output g_m[i,j], 1≤m≤M                      // g_m m-th good colony image, M # of good colonies on p[x,y]

   m=1
   for each s_n[x,y] s.t. c(n) = 1
       if IsGoodColony(p[x,y], e[x.y], s_n[x,y])
           g_m[i,j] = p.extract_colony_image(s_n[x,y])
           m++
       end if
   end for
   M=m-1
```

Level 2 – Isolated good colony presumptive identification:

```
Input g_m[i,j], 1 ≤ m ≤ M
Output pID(m), 1 ≤ m ≤ M

   for 1 ≤ m ≤ M
      compute pID[m] = CnnID (g_m[i,j])       // pID[m] presumptive identification vector ranking for g_m
   end for
```

Level 3 – Isolated colonies clustering by strains:

```
Input g_m[i,j], 1≤m≤M
Output sID[m], 1≤m≤M

   for 1 ≤ m ≤ M
      compute FV[: , m] = SCnnEmB (g_m[i,j])   // FV D×M matrix of D -dimensional feature vectors in the
                                               // embedding space computed for the M single good colonies
                                               // of the current plate
   end for
   compute cv = MeanShift (FV)                 // generate the proposed similarity-driven clustering where cv
                                               // is a vector of dimension M containing the cluster IDs
   C = cv.how_many_clusters
   for 1 ≤ c ≤ C
      cn[c] = cv.cluster_size(c)
      compute sID[i=find m s.t. cv[m]==c] = SmoothID (pID[i], cn[c])   // For the colonies belonging to the cluster c
                                                                       // updates pIDs to a common sID
   end for
```

Level 4 – Culture significance assessment and colony picking suggestions:

```
Input sID[m], e[x.y], plate_signif_rules(lab)
Output ps, cfu[min, max, s], mp[]

   compute cfu[min, max, s] = EstimateIDcount (e[x.y], sID[m])
   compute ps = PlateSignificance (cfu[min, max, s], plate_signif_rules(lab))
   for 1 ≤ s ≤ S
       if cfu[min, max, s].max != 0
           append mp[] ← Colony2PickIndex(p[x,y], e[x.y], sID[], s)   // List of best good colonies indexes to pick
       end if                                                         // for downstream tasks (Maldi-TOF,
AST,...)
   end for
```

Supplementary References

1. Ferrari, A., Lombardi, S., Signoroni, A. Bacterial colony counting with Convolutional Neural Networks in Digital Microbiology Imaging. *Pattern Recognit.* **61**, 629–640 (2017).
2. Nair, V., Hinton, G. E. Rectified linear units improve restricted boltzmann machines. in *Proc. of International Conference on Machine Learning (ICML'10)* **27**, 807–814 (2010).
3. Maas, A. L., Hannun, A. Y., Ng, A. Y. Rectifier nonlinearities improve neural network acoustic models. in *Proc. of International Conference on Machine Learning (ICML'13)* **30**, (2013).
4. He, K., Zhang, X., Ren, S., Sun, J. Delving deep into rectifiers: surpassing human-level performance on ImageNet classification. in *Proc. of IEEE International Conference on Computer Vision (ICCV'15)* 1026–1034 (2015).
5. Krizhevsky, A., Sutskever, I., Hinton, G.E. ImageNet Classification with Deep Convolutional Neural Networks. in *Proc. of Advances in Neural Information Processing Systems 25 (NIPS'12)* 1097–1105 (2012).
6. Hinton, G. E. *et al.* Improving neural networks by preventing co-adaptation of feature detectors. Preprint at https://arxiv.org/abs/1207.0580 (2012).
7. Glorot, X., Bengio, Y. Understanding the difficulty of training deep feedforward neural networks. in *Proc. of International Conference on Artificial Intelligence and Statistics (AISTATS'10)* 249–256 (2010).
8. Taigman, Y., Yang, M., Ranzato, M.'A., Wolf, L. DeepFace: closing the gap to human-level performance in face verification. in *Proc. of IEEE Conf. on Computer Vision and Pattern Recognition (CVPR'14)* 1701–1708 (2014).
9. Chopra, S., Hadsell, R. & LeCun, Y., Learning a similarity metric discriminatively, with application to face verification. in *Proc. of IEEE Conference on Computer Vision and Pattern Recognition (CVPR'05)* 539–546 (2005).
10. Kingma, D. P., Ba, J. Adam: A Method for Stochastic Optimization. in *Proc. of International Conference on Learning Representations (ICLR 2015)* (2015). Preprint at https://arxiv.org/abs/1412.6980
11. Rosenberg, A., Hirschberg, J. V-Measure: A conditional entropy-based external cluster evaluation measure. in *Proc. of Joint Conference on Empirical Methods in Natural Language Processing and Computational Natural Language Learning (EMNLP-CoNLL'07)* 410-420 (2007).
