# Supplementary material for: Hierarchical AI enables global interpretation of culture plates in the era of digital microbiology
Source: Nat Commun. 2023 Oct 28;14:6874. doi: 10.1038/s41467-023-42563-1 (PMC10613199; doi:10.1038/s41467-023-42563-1)
Supplement: Supplementary file 3 — Reporting Summary [file 41467_2023_42563_MOESM3_ESM.pdf]

nature portfolio

Corresponding author(s): Alberto Signoroni

Last updated by author(s): Oct 11, 2023

Reporting Summary

Nature Portfolio wishes to improve the reproducibility of the work that we publish. This form provides structure for consistency and transparency in reporting. For further information on Nature Portfolio policies, see our Editorial Policies and the Editorial Policy Checklist.

Statistics

For all statistical analyses, confirm that the following items are present in the figure legend, table legend, main text, or Methods section.

n/a	Confirmed	
☐	☒	The exact sample size (*n*) for each experimental group/condition, given as a discrete number and unit of measurement
☐	☒	A statement on whether measurements were taken from distinct samples or whether the same sample was measured repeatedly
☒	☐	The statistical test(s) used AND whether they are one- or two-sided<br>*Only common tests should be described solely by name; describe more complex techniques in the Methods section.*
☒	☐	A description of all covariates tested
☒	☐	A description of any assumptions or corrections, such as tests of normality and adjustment for multiple comparisons
☐	☒	A full description of the statistical parameters including central tendency (e.g. means) or other basic estimates (e.g. regression coefficient) AND variation (e.g. standard deviation) or associated estimates of uncertainty (e.g. confidence intervals)
☒	☐	For null hypothesis testing, the test statistic (e.g. *F*, *t*, *r*) with confidence intervals, effect sizes, degrees of freedom and *P* value noted<br>*Give P values as exact values whenever suitable.*
☒	☐	For Bayesian analysis, information on the choice of priors and Markov chain Monte Carlo settings
☒	☐	For hierarchical and complex designs, identification of the appropriate level for tests and full reporting of outcomes
☒	☐	Estimates of effect sizes (e.g. Cohen's *d*, Pearson's *r*), indicating how they were calculated

Our web collection on statistics for biologists contains articles on many of the points above.

Software and code

Policy information about availability of computer code

Data collection	Both the colony-level and the plate-level datasets come from hi-resolution digital scans of cultured plates from clinical specimens processed by WaspLabTM (by Copan WASP , Italy) full laboratory automation facilities.
Data analysis	A detailed pseudocode has been provided in the Supplementary Information file. The image analysis code (architecture and trained models) is not publicly available due to some proprietary restrictions. They can be made available from the authors on request and with permission of Copan WASP. Excel data sheets (ver. 16) were used for agreement and statistical analyses. Confusion matrices and diagrams were created with Keynote (ver.11) and Blender (ver.2.8).

For manuscripts utilizing custom algorithms or software that are central to the research but not yet described in published literature, software must be made available to editors and reviewers. We strongly encourage code deposition in a community repository (e.g. GitHub). See the Nature Portfolio guidelines for submitting code & software for further information.

Data

Policy information about availability of data

All manuscripts must include a data availability statement. This statement should provide the following information, where applicable:

- Accession codes, unique identifiers, or web links for publicly available datasets
- A description of any restrictions on data availability
- For clinical datasets or third party data, please ensure that the statement adheres to our policy

All data supporting the findings described in this manuscript are available in the article and in the Supplementary Information. Source data for all data figures are provided with this paper. The core colony image dataset is available on Figshare with the following identifier (https://doi.org/10.6084/m9.figshare.24203961). The use-case plate dataset is property of Tricore Laboratories (Albuquerque, New Mexico, USA), it was used under license for the current study and is not publicly available. Requests can be addressed to the corresponding author (expected response time 2 weeks) and access will require explicit permission from Tricore Labs.

Human research participants

Policy information about studies involving human research participants and Sex and Gender in Research.

Reporting on sex and gender	n/a
Population characteristics	n/a
Recruitment	n/a
Ethics oversight	n/a

Note that full information on the approval of the study protocol must also be provided in the manuscript.

Field-specific reporting

Please select the one below that is the best fit for your research. If you are not sure, read the appropriate sections before making your selection.

☐ Life sciences ☐ Behavioural & social sciences ☒ Ecological, evolutionary & environmental sciences

For a reference copy of the document with all sections, see nature.com/documents/nr-reporting-summary-flat.pdf

Ecological, evolutionary & environmental sciences study design

All studies must disclose on these points even when the disclosure is negative.

Study description	Aiming at demonstrating the possibility of supporting, by AI-driven methods, the difficult and massive tasks related to culture plate interpretation, in their full clinical complexity, the first innovative study in this direction is proposed here, where a hierarchical multi-network DL-based architecture operates on an integral flow of digitized diagnostic culture plates generated by modern full clinical microbiology laboratory automation.
Research sample	The research sample consists in 2 datasets: 1) Colony-level and 2) Plate-level dataset. Both the datasets represents bacterial growth on blood agar plates.<br>1) Colony-level dataset: from 1,351 pure flora plate images (1,321 from clinical specimens and 30 from ATCC), 26,213 isolated colony images (24,781 clinical and 1,432 from ATCC) were extracted. The colony-level dataset provides a broad representation of the growth morphotypes on blood agar for each of the 32 species considered. This dataset is used to train and test the bacterial species recognition models presented in this work.<br>2) Plate-level dataset: 5,051 images (from the same number of physical plates) taken from cultures performed at TriCore Reference Laboratories in Albuquerque, NM (US). This dataset is used to test the entire analysis architecture proposed in this work, which enables the complete clinical interpretation of the culture plates.
Sampling strategy	1) From the network training point of view more than 26,000 single colony images allowed to well cover the morphologic variabilities of 32 bacterial species (and 16 phylogenic groups) in standardized culturing conditions guaranteed by full laboratory automation systems. While each species/group was sufficiently well represented, more common pathogens were naturally represented with more samples. Despite on purpose addition of less commonly encountered pathogens, possible skewed class effects were monitored and acknowledged in the analysis and were verified to be not detrimental.<br>2) The whole clinical flow of plate images acquired non-stop in one entire week of laboratory workflow (for a total of more than 5,000 plates). This was considered highly representative of the typical clinical variability of UTI samples and allowed statistically significant human/machine agreement analysis.
Data collection	Both the colony-level and the plate-level datasets come from hi-resolution digital scans of cultured plates from clinical specimens processed by WaspLab (by Copan WASP, Italy) FLA facilities. The colony-level collection was curated by the authors A.F. and S.L. and ground truth association between images and species was derived in the majority of cases from MALDI-ToF bacteria identification of

	clinical samples and in a smaller fraction from prior knowledge (ATCC samples), in the proportions described before. The collection of laboratory plates took place under the responsibility and supervision of the author K.C.. Images of both datasets was produced by WaspLabTM Laboratory Automation plants (Copan WASP, Italy), where 16Mpixel tri-linear colour camera is used to produce hi-res digital images by pushbroom line-scanning under a calibrated white LED lighting system. Combined with high-quality telecentric optics, this guarantees low geometric distortion and high spatial resolution (24.5 µm/pixel). All the plates were inoculated from urine clinical specimens using a 1µl loop on REMELTM blood agar and incubated from 17 to 23 hours in CO2 at 37°C.
Timing and spatial scale	There are no timing and spatial scale dependencies on how bacteria species grows on culturing media. The Clinical digital plates were acquired continuously in fall 2017 during an entire week of operation on UTI plates in a large clinical microbiology laboratory (TriCore Reference Laboratories, Albuquerque, New Mexico, US).
Data exclusions	No data were excluded from the plate interpretation analyses
Reproducibility	Very stable experimental condition are guaranteed by the use of highly standardized (well over manual procedures) processing guaranteed by the full laboratory automation used to process and digitize the samples (WaspLab, Copan).
Randomization	For dataset 1) ground truth labeling is guaranteed by the use of pure flora cultures to acquire single colony images and no discriminative selection of isolated colonies on the plates was made to build the dataset. Subdivision in training/validating/testing sets in the 60/20/20 proportions was performed at the plate level with random selection among each of the 32 represented species, in order to avoid having colonies from the same plate appearing in different portions of the colony-level image dataset.<br>Not relevant to dataset 2) since the whole flow of UTI samples was inserted without subgrouping.
Blinding	For dataset 1) the colony-level clinical dataset was created from cultured plates of pure flora cultures collected in a fully anonymised form, without any kind of interaction or interference with diagnostic/prognostic/therapeutic processes, and no additional information affects analysis performed by the system.<br>For dataset 2) the specialists who categorized clinical plate images were completely unaware of what was performed in parallel by the machine and results have been analysed a posteriori without any effect on the laboratory diagnostic workflow.

Did the study involve field work? ☐ Yes ☒ No

Reporting for specific materials, systems and methods

We require information from authors about some types of materials, experimental systems and methods used in many studies. Here, indicate whether each material, system or method listed is relevant to your study. If you are not sure if a list item applies to your research, read the appropriate section before selecting a response.

Materials & experimental systems

n/a	Involved in the study
☒	☐ Antibodies
☒	☐ Eukaryotic cell lines
☒	☐ Palaeontology and archaeology
☐	☒ Animals and other organisms
☒	☐ Clinical data
☒	☐ Dual use research of concern

Methods

n/a	Involved in the study
☒	☐ ChIP-seq
☒	☐ Flow cytometry
☒	☐ MRI-based neuroimaging

Animals and other research organisms

Policy information about studies involving animals; ARRIVE guidelines recommended for reporting animal research, and Sex and Gender in Research

Laboratory animals	Study did not involved laboratory or any animals, but only microrganisms.
Wild animals	The study did not involved wild animals.
Reporting on sex	Not applicable
Field-collected samples	The study involved microrganisms on cultured plates from clinical specimens processed by WaspLab™ (by Copan WASP®, Italy) full laboratory automation facilities. The colony-level clinical dataset was created from cultured plates acquired in collaborating labs in the US, and collected in a fully anonymised form, without any kind of interaction or interference with the laboratory and patient diagnostic/prognostic/therapeutic processes. The plate-level clinical dataset is made of 5051 WaspLab™ images (from same number of plates) taken from cultures performed at TriCore Reference Laboratories in Albuquerque, NM (US)
Ethics oversight	No ethical approval or guidance was required for microrganisms study.

Note that full information on the approval of the study protocol must also be provided in the manuscript.
